# Influence of Pharmacological Agents on Orthodontic Tooth Movement: A Systematic Review

**DOI:** 10.3390/bioengineering13020224

**Published:** 2026-02-14

**Authors:** Lucia Giannini, Federica Macrì, Angelo Michele Inchingolo, Francesco Inchingolo, Gianna Dipalma, Cinzia Maspero

**Affiliations:** 1 Fondazione IRCCS Cà Granda Ospedale Maggiore Policlinico, 20122 Milan, Italy; federica.macri@unimi.it (F.M.); cinzia.maspero@unimi.it (C.M.); 2 Department of Biomedical, Surgical and Dental Sciences, University of Milan, 20122 Milan, Italy; angelomichele.inchingolo@uniba.it (A.M.I.); g.dipalma@unilink.it (G.D.); 3 Department of Interdisciplinary Medicine, University of Bari Aldo Moro, 70124 Bari, Italy; 4 Department of Life Science, Health and Health Professional, Link Campus University, 00165 Roma, Italy

**Keywords:** bone remodeling, non-steroidal anti-inflammatory drugs, orthodontic pain, orthodontic tooth movement, periodontal response, pharmacological agents, systematic review

## Abstract

Background: Pharmacological agents may interfere with the biological processes underlying orthodontic tooth movement (OTM), potentially affecting treatment duration, pain control, and periodontal outcomes. Methods: A systematic review was conducted according to PRISMA 2020 guidelines and registered in PROSPERO. Human studies were prioritized to assess clinically relevant effects on OTM and pain, while animal and in vitro studies were included to support biological interpretation. Results: Sixty-four studies were included. Human evidence indicates that NSAIDs effectively reduce orthodontic pain but may decrease the rate of tooth movement in a dose-dependent manner. Antiresorptive drugs, particularly bisphosphonates, were consistently associated with reduced OTM. Topical antimicrobials, fluoride agents, and probiotics improved periodontal and enamel outcomes without significantly affecting tooth movement. Most evidence derived from preclinical models showed mechanistic consistency but limited clinical applicability. Overall certainty of evidence ranged from low to very low. Conclusions: Pharmacological agents can influence orthodontic outcomes, particularly pain perception and tooth movement rate. A thorough medication history is essential during orthodontic treatment planning. Current evidence remains limited, highlighting the need for well-designed clinical trials to support personalized orthodontic care.

## 1. Introduction

Orthodontic tooth movement (OTM) is a biologically driven process resulting from the application of controlled mechanical forces to teeth, leading to coordinated inflammatory responses and alveolar bone remodeling [1,2,3,4]. These events involve the activation of periodontal ligament cells, the release of cytokines and prostaglandins, and the regulation of osteoclastic and osteoblastic activity. As a result, any factor capable of modulating inflammation or bone metabolism has the potential to influence the rate and pattern of tooth movement [5,6,7,8].

In contemporary orthodontic practice, an increasing number of patients—both adolescents and adults—are exposed to systemic or local pharmacological agents for acute or chronic conditions. Analgesics, anti-inflammatory drugs, bone metabolism–modifying agents, hormones, and immunomodulatory therapies are frequently prescribed and may interfere with the biological mechanisms underlying OTM. From a clinical perspective, such interactions may affect treatment duration, anchorage control, pain management, and the risk of adverse effects such as root resorption or periodontal alterations [9,10,11,12].

Experimental and clinical studies have reported heterogeneous and sometimes conflicting effects of pharmacological agents on OTM. Non-steroidal anti-inflammatory drugs (NSAIDs) have been shown to reduce orthodontic pain but may slow tooth movement by inhibiting prostaglandin-mediated bone resorption. Conversely, agents influencing bone turnover, such as parathyroid hormone or vitamin D, have been associated with accelerated movement in preclinical models. However, much of the available evidence derives from animal or in vitro studies, and the translational relevance to human orthodontic treatment remains uncertain [13,14,15,16,17,18].

Previous reviews have addressed selected drug classes or specific outcomes, but a focused synthesis integrating clinically relevant outcomes with underlying biological mechanisms is still lacking. Importantly, the wide variability in study design, experimental models, and outcome measures has contributed to fragmented knowledge and limited clinical applicability.

Therefore, the aim of this systematic review is to address the following focused question: how do pharmacological agents influence orthodontic tooth movement and its underlying biological mechanisms?

Human studies were prioritized to assess clinical outcomes such as tooth movement rate, pain, and treatment-related effects, while animal and in vitro studies were included to elucidate mechanistic pathways supporting or explaining clinical observations. By adopting a translational framework, this review seeks to provide a critical and clinically oriented synthesis of the evidence, while clearly acknowledging its methodological limitations.

## 2. Materials and Methods

### 2.1. Protocol

This systematic review was conducted in accordance with the PRISMA 2020 and PRISMA-P guidelines for transparent reporting of systematic reviews.

The protocol of this systematic review was prospectively registered in PROSPERO [CRD420251242400].

### 2.2. Review Objectives and Hypotheses

The primary objective of this review was to critically evaluate the effects of pharmacological agents on orthodontic treatment. Specifically, the review investigated how different drugs influence.

The analysis focused on how different drugs influence:

- Orthodontic tooth movement;
- Bone remodeling and inflammatory markers;
- Periodontal and pulpal responses;
- Pain modulation during orthodontic treatment;
- Enamel demineralization;
- Clinical implications for orthodontic mechanics and treatment planning in growing patients and adults.

Underlying hypotheses were that:

- NSAIDs and other anti-inflammatory drugs reduce OTM by suppressing prostaglandin-mediated bone resorption.
- Bone metabolism-modifying agents can impact the rate and pattern of tooth movement.
- Hormonal agents and immunomodulatory drugs alter periodontal tissue responses.
- Topical agents influence enamel stability during fixed appliance therapy.

### 2.3. PICO

**Population (P):** Patients undergoing orthodontic treatment; animal and in vitro models were included to support biological interpretation.
**Intervention (I):** Systemic or local pharmacological agents administered during orthodontic treatment.
**Comparison (C):** No pharmacological exposure, placebo, or alternative drug when applicable.
**Outcomes (O):**

- **Primary outcome:** Rate or extent of orthodontic tooth movement.
- **Secondary outcomes:** Pain perception during orthodontic treatment and periodontal or enamel responses directly related to appliance therapy.

Mechanistic outcomes derived from animal or in vitro studies (e.g., inflammatory mediators and bone remodeling markers) were considered supportive and hypothesis-generating.

### 2.4. Search Strategy and Study Selection

A systematic search was conducted in PubMed-MEDLINE and Scopus databases. A manual search of reference lists of included articles and relevant reviews was performed. Grey literature was searched through OpenGrey and Google Scholar and reference lists of included studies and relevant reviews were manually searched.

The search included studies published between January 2000 and December 2025, in English.

A structured search strategy using MeSH terms and free-text keywords was applied.

***PubMed search:***

[[“orthodontic tooth movement”[tiab] OR “orthodontic movement”[tiab] OR “tooth movement”[tiab] OR orthodontic*[tiab]] AND [“Pharmaceutical Preparations”[MeSH] OR “Drug Therapy”[MeSH] OR drug*[tiab] OR medication*[tiab] OR pharmacolog*[tiab] OR medicine*[tiab] OR analgesic*[tiab] OR “anti-inflammatory”[tiab] OR corticosteroid*[tiab] OR bisphosphonate*[tiab] OR hormone*[tiab] OR prostaglandin*[tiab] OR “Anti-Inflammatory Agents, Non-Steroidal”[MeSH] OR “Bone Remodeling”[MeSH]] AND [“bone remodeling”[tiab] OR “bone resorption”[tiab] OR “orthodontic force”[tiab] OR “tooth movement”[tiab]] AND Humans[MeSH Terms] AND English[lang] NOT [review[Publication Type] OR case reports[Publication Type]] AND [“1 January 2000“[Date-Publication]: “31 December 2025”[Date-Publication]]

***Scopus search:***

*TITLE-ABS-KEY [“orthodontic tooth movement” OR “orthodontic treatment” OR “tooth movement” OR “orthodontic therapy”] AND [“drug therapy” OR drug OR medication OR NSAID OR bisphosphonate OR corticosteroid OR antibiotic OR statin OR metformin OR hormone OR analgesic OR chlorhexidine] AND [“effect” OR “influence” OR “impact” OR “interaction” OR “response” OR “tooth movement rate” OR “anchorage loss” OR “bone remodeling” OR “bone resorption”] AND [child OR children OR pediatric OR paediatric OR adolescent OR “growing patient”] AND NOT [“implant” OR “cleft” OR “craniofacial” OR “surgery”] AND PUBYEAR > 1999 AND PUBYEAR < 2026 AND [LIMIT-TO [DOCTYPE, “ar”] AND [LIMIT-TO [LANGUAGE, “English”]*

The last search was performed on 20 October 2025.

### 2.5. Study Selection

The study selection process was conducted independently by two reviewers. Any discrepancies in the screening or eligibility assessment were resolved through discussion and consensus.

All retrieved citations from PubMed-MEDLINE, Scopus, and grey literature were imported into Microsoft Excel, and duplicate records were removed prior to screening.

The screening process was conducted in two sequential phases: initial screening of titles and abstracts, followed by full-text assessment for eligibility.

A PRISMA flow diagram illustrates the selection process.

### 2.6. Inclusion and Exclusion Criteria

Inclusion criteria:

- Randomized controlled trials [RCTs], controlled clinical trials, observational studies, in vitro studies, and preclinical animal studies;
- Studies evaluating the effects of drugs on tooth movement, bone remodelling, periodontal response, or pain modulation during orthodontic treatment;
- Published between 2000 and 2025;
- Full text available in English.

Exclusion criteria:

- Studies not relevant to the review objective;
- Studies on dental topics unrelated to orthodontics;
- Case reports, letters, or abstracts;
- Non-English articles without full text

Studies focused exclusively on surgical orthodontics or craniofacial anomalies

### 2.7. Data Extraction and Organization

Data extraction was conducted independently by two reviewers using a standardized and piloted extraction sheet.

For each included study, the following data were collected:

- Authors and year of publication;
- Study design;
- Population or experimental model;
- Drug studied;
- Orthodontic intervention or biological outcome;
- Main results;
- Clinical relevance of drug effects.

To facilitate interpretation, studies were organized by drug categories:

1 NSAIDs and analgesics;
2 Drugs affecting bone metabolism [bisphosphonates, RANK/RANKL modulators, vitamin D, etc.];
3 Hormones and endocrine agents;
4 Antiseptics and antimicrobials;
5 Special systemic or immunomodulatory drugs;
6 Other drugs with potential orthodontic impact.

Given the overlap in clinical purpose and outcomes, antiseptics, antimicrobials, probiotics, and preventive topical agents were analyzed within a single category focusing on periodontal and enamel protection during orthodontic treatment.

This classification allows for a clearer understanding of the specific interaction of each drug with orthodontic treatments.

### 2.8. Risk of Bias Assessment and GRADE

Risk of bias was independently assessed by two reviewers using validated tools according to study design:

RoB2 for randomized controlled trials;

ROBINS-I for non-randomized clinical studies;

SYRCLE’s RoB tool for animal studies;

OHAT risk-of-bias tool for in vitro studies;

Disagreements were resolved through discussion.

The certainty of evidence for each outcome category was evaluated using the GRADE approach, considering risk of bias, inconsistency, indirectness, imprecision, and publication bias.

For each included study, the overall risk-of-bias judgement was supported by explicit methodological reasons, which are reported in the risk-of-bias tables and Supplementary Material.

### 2.9. Data Analysis

Due to clinical and methodological heterogeneity in drugs, experimental models, and measured outcomes, a meta-analysis was not performed. A qualitative synthesis of the studies was conducted using a tabular comparison. Where available, quantitative results such as tooth movement rates, inflammatory biomarker levels, or tissue effects were reported and compared. Descriptive statistics were used to summarize the distribution of studies by drug type and outcome.

## 3. Results

**Figure 1:**
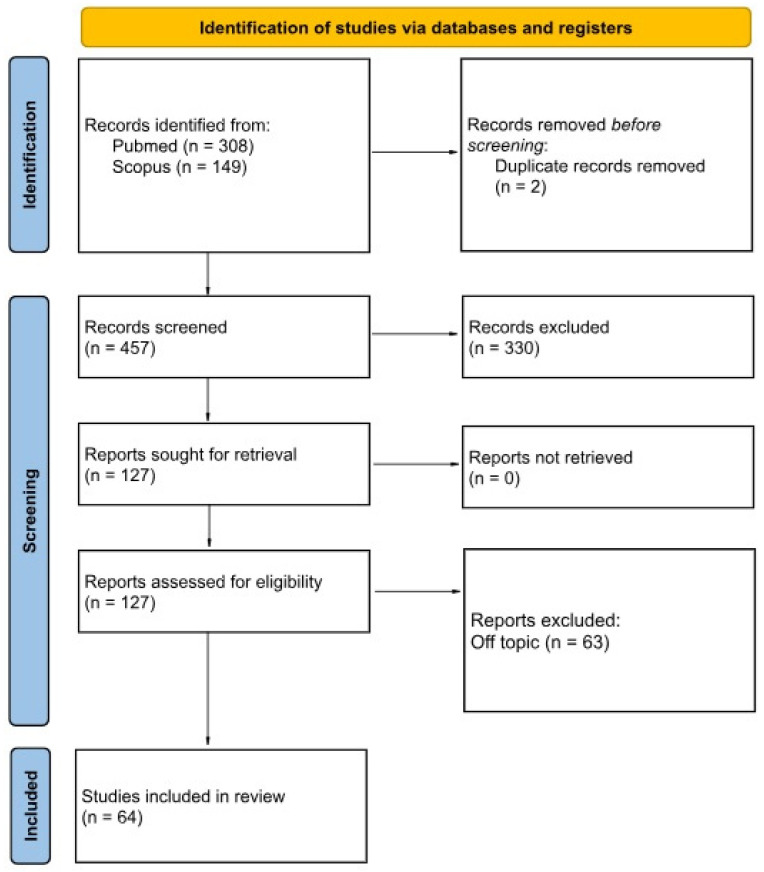
PRISMA 2020 flow diagram illustrating the study identification, screening, eligibility assessment and inclusion process.

The electronic and manual searches identified a total of 457 records. After removal of duplicates and two-stage screening (title/abstract and full-text assessment), 64 studies met the inclusion criteria and were included in the qualitative synthesis. The study selection process is summarized in the PRISMA flow diagram (Figure 1).

### 3.1. Characteristics of Included Studies

The included studies comprised a heterogeneous body of evidence, including randomized controlled trials, non-randomized clinical studies, observational studies, animal models, and in vitro experiments. Pharmacological agents investigated encompassed NSAIDs and analgesics, drugs affecting bone metabolism, hormonal and endocrine agents, antimicrobials, probiotics, and selected immunomodulatory drugs.

Given this heterogeneity, results were synthesized qualitatively and organized according to drug class and clinical relevance. Detailed characteristics of individual studies are reported in the Supplementary Materials.

### 3.2. Human Clinical Evidence on Orthodontic Tooth Movement and Pain

#### 3.2.1. NSAIDs and Analgesics

Human clinical studies consistently demonstrated that NSAIDs effectively reduce orthodontic pain, particularly during the early phases of treatment. Ibuprofen showed superior analgesic efficacy compared with acetaminophen in several randomized trials, both when administered pre-emptively and post-activation. Acetaminophen also reduced pain intensity but generally to a lesser extent [18,19,20].

Evidence from animal models suggested that NSAIDs, including ibuprofen and meloxicam, may reduce the rate of orthodontic tooth movement (OTM) through inhibition of prostaglandin-mediated osteoclastic activity. However, this inhibitory effect was primarily supported by preclinical data, while direct clinical evidence on OTM reduction in humans remains limited [21,22]. (Tables S1–S3).

Collectively, these findings indicate that NSAIDs provide effective pain control but may influence bone remodeling mechanisms involved in OTM, particularly with prolonged or high-dose administration. Summary of clinically relevant findings is reported in Table 1.

#### 3.2.2. Drugs Affecting Bone Metabolism

Drugs modulating bone remodeling showed consistent effects on OTM, with direction dependent on their mechanism of action [23,24,25].

Antiresorptive agents, particularly bisphosphonates, were associated with reduced tooth movement in both human observational studies and animal models. Patients undergoing chronic bisphosphonate therapy demonstrated slower orthodontic movement and increased difficulty in achieving planned tooth displacement.

Conversely, intermittent parathyroid hormone (PTH) administration and prostaglandin E1 (PGE1) were associated with accelerated OTM, particularly in experimental settings or in conjunction with surgical interventions. However, human clinical evidence supporting routine clinical use of these agents remains limited [26,27,28,29,30,31,32,33].

Other agents, such as simvastatin, showed a modulatory effect by preserving anchorage without significantly altering OTM. Tables S4–S6.

A clinically oriented synthesis of these effects is provided in Table 2, while detailed mechanistic and preclinical data are reported in the Supplementary Materials.

#### 3.2.3. Hormonal and Endocrine Agents

Hormonal status and endocrine agents were associated with variable modulation of OTM. Clinical studies suggested that sex hormones may influence inter-individual variability in tooth movement, with testosterone associated with faster OTM and estrogen exerting a modulatory effect. Tables S7–S9.

Vitamin D and vitamin E supplementation showed potential to support bone remodeling and antioxidant balance, with limited evidence suggesting a possible acceleration of OTM. In contrast, glucocorticoid therapy and nicotine exposure were associated with impaired bone metabolism and potentially slower tooth movement.

Given the predominance of observational and preclinical data, these findings should be interpreted cautiously. Detailed results are reported in the Supplementary Materials [34,35,36,37,38,39,40,41].

#### 3.2.4. Periodontal and Enamel Outcomes in Orthodontic Patients

Topical antimicrobials and preventive agents demonstrated consistent benefits for periodontal and enamel health without significantly affecting OTM.

Chlorhexidine mouthwashes and varnishes effectively reduced plaque accumulation and bacterial load, particularly Streptococcus mutans, although prolonged use was associated with side effects such as tooth staining and taste alteration. Probiotics, fluoride formulations, MI Paste Plus, and natural dentifrices improved gingival indices and reduced enamel demineralization around orthodontic appliances. Tables S10–S12.

A summary of clinically relevant periodontal and enamel outcomes is provided in Table 3. Detailed comparisons of formulations and protocols are reported in the Supplementary Materials [42,43,44,45,46,47,48,49,50,51,52,53,54,55,56,57,58,59,60,61,62,63,64,65].

#### 3.2.5. Special Systemic Drugs and Immunomodulators

Limited evidence suggested that certain systemic drugs, including cyclosporine, methylphenidate, and long-term corticosteroids, may adversely affect periodontal tissues, bone remodeling, or craniofacial growth, potentially complicating orthodontic treatment. Tables S13–S17.

Conversely, thymosin α1 showed beneficial effects on periodontal healing in specific clinical contexts, such as tooth reimplantation. Due to the limited number of studies and high risk of bias, these findings should be considered hypothesis-generating [66,67,68,69,70,71,72,73,74].

### 3.3. Risk of Bias and Certainty of Evidence

Overall, most included studies demonstrated moderate to high risk of bias, primarily due to inadequate randomization, lack of blinding, and small sample sizes. Using the GRADE approach, the certainty of evidence was rated as low to very low across most outcomes. A condensed GRADE summary is presented in Table 4 and Table 5, while detailed assessments are provided in the Supplementary Materials.

## 4. Discussion

This systematic review evaluated the effects of systemic and local pharmacological agents on orthodontic tooth movement and related biological and clinical outcomes. The available evidence suggests that several commonly prescribed drugs can influence orthodontic treatment primarily through modulation of inflammatory responses and bone remodeling processes [1,2,3,4,5]. However, the direction and clinical relevance of these effects vary considerably depending on drug class, dosage, duration of administration, and patient-related factors (Table 6).

One of the most consistent findings concerns the use of non-steroidal anti-inflammatory drugs (NSAIDs) during orthodontic treatment. Human clinical studies confirmed that NSAIDs are effective in reducing orthodontic pain, particularly during the early phases following appliance activation [1,2,17,18,19,20]. Ibuprofen was generally more effective than acetaminophen in pain control, especially in the first 24–48 h [2,17,18,20]. At the same time, experimental evidence indicates that NSAIDs may reduce the rate of orthodontic tooth movement by inhibiting prostaglandin-mediated osteoclastic activity [5,21,22]. Although this inhibitory effect on tooth movement has been demonstrated mainly in animal models, it raises relevant clinical considerations, particularly in patients requiring prolonged or frequent analgesic use. From a clinical perspective, careful selection of analgesics remains important to balance effective pain management with preservation of orthodontic efficiency [6,7,8].

Drugs affecting bone metabolism showed more pronounced and predictable effects on orthodontic tooth movement. Antiresorptive agents, such as bisphosphonates, were consistently associated with reduced tooth movement in both clinical and experimental studies [27,28]. In patients undergoing long-term bisphosphonate therapy, orthodontic treatment may be characterized by a potentially slower response, increased resistance to movement, and a higher risk of suboptimal outcomes [27]. Conversely, agents that stimulate bone remodeling, including intermittent parathyroid hormone and prostaglandin E1, demonstrated a potential to accelerate orthodontic tooth movement, particularly in experimental settings or in conjunction with surgical interventions [9,25,26,31,32]. Nevertheless, the limited availability of controlled clinical trials and concerns regarding adverse effects, such as root resorption, currently restrict their routine clinical application [31,32].

Hormonal and endocrine agents appeared to contribute to inter-individual variability in orthodontic response rather than exerting uniform effects. Clinical and experimental data suggest that sex hormones may modulate bone turnover and periodontal ligament response, potentially influencing the rate of tooth movement [11,40]. Similarly, vitamin D and vitamin E supplementation were associated with favorable effects on bone metabolism and oxidative stress balance, with limited evidence suggesting a possible supportive role during orthodontic treatment [34,35,36,37]. In contrast, chronic glucocorticoid therapy and nicotine exposure were generally associated with impaired bone quality and altered tissue responses, potentially complicating orthodontic mechanics and treatment duration [33,38,39]. These findings highlight the importance of considering endocrine status and lifestyle factors during treatment planning.

In contrast to systemic drugs affecting bone remodeling, topical agents and microbiome-modulating therapies primarily influenced periodontal and enamel outcomes rather than orthodontic tooth movement. Chlorhexidine-based products consistently reduced plaque accumulation and bacterial load around orthodontic appliances [42,43,44,45,46,47,48], although their prolonged use was associated with well-known side effects, including tooth staining and taste alteration [42,47]. Probiotics, fluoride-based products, remineralizing agents, and natural dentifrices demonstrated beneficial effects on gingival health and enamel demineralization without significantly affecting tooth movement [13,49,52,53,54,55,56,57,58,59,60,61,62,63,64,65]. These interventions appear to represent valuable adjuncts for maintaining oral health during fixed appliance therapy, particularly in patients at increased risk of caries or periodontal inflammation.

Special systemic drugs and immunomodulatory agents were supported by limited and heterogeneous evidence. Immunosuppressive therapies, including cyclosporine and long-term corticosteroids, were associated with increased risk of gingival overgrowth, periodontal complications, and altered craniofacial growth patterns [12,67,70]. Conversely, thymosin α1 showed beneficial effects on periodontal healing in specific clinical scenarios, such as tooth reimplantation [66]. Due to the limited number of studies and the predominance of observational or experimental designs, these findings should be interpreted cautiously and primarily serve to raise clinical awareness rather than guide definitive treatment decisions [68].

Unlike previous reviews focusing on single drug classes, this review integrates systemic, topical and microbiome-modulating agents within a translational orthodontic framework.

A relevant aspect of this review is the inclusion of animal and in vitro studies to support biological interpretation of clinical findings. While preclinical models provide valuable insights into the mechanisms underlying orthodontic tooth movement, their direct translation to clinical practice is limited by differences in force application, tissue response, and systemic conditions [72,73,74]. For this reason, preclinical evidence in the present review was considered supportive rather than determinant for clinical recommendations. 

Several limitations must be acknowledged. The overall quality of the available evidence was low to very low, with many studies affected by high risk of bias, small sample sizes, and methodological heterogeneity [14,15]. The frequent reliance on animal models and surrogate biochemical markers further limits the direct applicability of findings to human orthodontic treatment. In addition, differences in drug dosage, administration protocols, and outcome measures prevented quantitative synthesis and meta-analysis.

Despite these limitations, the present review highlights the clinical relevance of pharmacological factors in orthodontic treatment. A thorough medication history should be considered an essential component of orthodontic diagnosis and treatment planning. Awareness of potential drug–orthodontic interactions may help clinicians anticipate variations in tooth movement, adjust biomechanics, and optimize patient management [16]. Future well-designed clinical studies are required to clarify drug-specific effects and to support evidence-based, personalized orthodontic care [75,76,77,78,79,80,81,82,83,84].

## 5. Conclusions

This systematic review highlights that pharmacological agents may influence orthodontic tooth movement primarily through modulation of inflammation and bone remodeling. Clinical evidence from human studies supports effective pain reduction with NSAIDs, although potential effects on tooth movement rate should be considered. Antiresorptive drugs are consistently associated with slower orthodontic movement, while topical preventive agents improve periodontal and enamel health without altering OTM [85,86,87,88,89].

Most mechanistic insights derive from animal and in vitro studies and should be interpreted cautiously when applied to clinical practice. Given the overall low certainty of available evidence, clinicians should individualize orthodontic treatment based on comprehensive medication history and risk assessment. Further high-quality human studies are required to clarify drug-specific effects and guide evidence-based orthodontic decision-making [90,91,92,93,94,95,96,97,98,99,100,101,102].

## Figures and Tables

**Table 1:** Effects of pharmacological agents on orthodontic tooth movement (OTM).

Drug Category	Main Agents	Effect on OTM	Evidence Source	Overall Risk of Bias	Interpretation
NSAIDs/analgesics	Ibuprofen, meloxicam, aspirin	↓ or ↔	Human, animal	Moderate–High	May slow OTM by inhibiting prostaglandin-mediated bone resorption
Antiresorptive drugs	Bisphosphonates, OPG	↓	Human, animal	High	Consistent inhibition of OTM due to reduced osteoclast activity
Bone turnover enhancers	PTH analogues, prostaglandins	↑	Animal, limited human	High	Acceleration observed mainly in preclinical models
Hormonal/endocrine agents	Vitamin D, vitamin E, sex hormones	↑/variable	Animal, observational	High	Potential modulation of bone remodeling; high interstudy variability
Immunomodulatory/special drugs	Methylphenidate, cyclosporine	↓/variable	Animal, observational	High	Limited and heterogeneous evidence
Topical/preventive agents	Chlorhexidine, fluoride, probiotics	↔	Human	Moderate	No direct effect on OTM

Abbreviations: ↑ acceleration; ↓ inhibition; ↔ no consistent effect; OTM = orthodontic tooth movement.

**Table 2:** Effects of pharmacological agents on pain and inflammatory response during orthodontic treatment.

Drug Category	Main Agents	Effect on Pain	Effect on Inflammation	Evidence Source	Overall Risk of Bias	Clinical Relevance
NSAIDs	Ibuprofen, meloxicam	↓↓	↓	Human, animal	Moderate	Effective pain control; possible impact on OTM
Analgesics (non-NSAID)	Acetaminophen	↓	↔	Human	Moderate	Pain relief without clear effect on OTM
Hormonal/antioxidant agents	Vitamin E	↓	↓ oxidative stress	Human, animal	High	Supportive role; limited clinical data
Topical agents	Benzocaine wax, ketoprofen gel	↓ (local)	↔	Human	Moderate	Adjunctive mucosal pain relief
Immunomodulators	Thymosin α1	↓	↓ cytokine expression	Human (limited)	Moderate–High	Exploratory evidence only

**Abbreviations:** ↓ inhibition; ↔ no consistent effect; OTM = orthodontic tooth movement.

**Table 3:** Effects of pharmacological and topical agents on periodontal and enamel health.

Agent Category	Main Agents	Periodontal Effects	Enamel Effects	Evidence Source	Overall Risk of Bias	Clinical Implication
Antiseptics	Chlorhexidine	↓ plaque, ↓ gingival inflammation	↔	Human	Moderate–High	Effective adjunct; monitor side effects
Fluoride-based agents	Fluoride varnish, AmF/SnF_2_	↓ gingivitis	↓ demineralization	Human	Moderate	Preventive benefit during fixed appliances
Probiotics	Lactobacillus spp.	↓ plaque acidogenicity	↔	Human	Moderate	Supportive role in oral hygiene
Remineralizing agents	MI Paste Plus, CPP-ACP	↔	↑ remineralization	Human	Low–Moderate	Enamel protection post-treatment
Oxidizing agents	H_2_O_2_ rinses	↓ gingival inflammation	↔	Human	High	Adjunctive, short-term use

**Abbreviations:** ↑ acceleration; ↓ inhibition; ↔ no consistent effect; OTM = orthodontic tooth movement.

**Table 4:** Outcome: effects of drugs on tooth movement.

GRADE Domain	Judgment	Explanation
Risk of bias	Very serious ↓↓	Most studies showed a high risk of bias due to inadequate randomization, lack of allocation concealment, no blinding, and incomplete reporting.
Inconsistency	Serious ↓	Substantial heterogeneity across drug categories, study designs, models, and outcome measures.
Indirectness	Very serious ↓↓	Many studies used animal models limiting applicability to clinical orthodontic movement.
Imprecision	Serious ↓	Small sample sizes, short follow-up periods and high variability reduced precision of effect estimates.
Publication bias	Possible ↓	Inconsistent reporting, lack of preregistered protocols and potential selective publication.
Overall certainty	Very low	The true effect of pharmacological agents on orthodontic tooth movement is likely to differ substantially from current estimates.

**Table 5:** Integrated Interpretation Across All Drug Classes.

Category	Findings	Examples/Notes
1. Effects on Orthodontic Tooth Movement [OTM]	Accelerators	PTH, PGE1, Vitamin D
	Inhibitors	Bisphosphonates, GLP-1 agonists, Estrogen, NSAIDs [dose-dependent]
	Neutral/Modulators	Simvastatin, Probiotics, Antimicrobial agents
2. Effects on Periodontal and Pulpal Health	Protective	Chlorhexidine varnish, Probiotics, Red propolis, Vitamin E [antioxidant]
	Risk-enhancing	IL-17 [root resorption], Nicotine [reduced osteogenesis], Glucocorticoids [immunosuppression]
3. Methodological Heterogeneity	Limitations of available evidence	Variability in dosage, administration route, follow-up, and experimental model [human vs. animal] reduces comparability across studies

**Table 6:** Overall Clinical Implications of Pharmacological Agents in Orthodontics.

Clinical Domain	Key Implications	Examples/Notes
**Medication history**	Must be routinely assessed as part of orthodontic diagnosis	Essential as noted by van Venrooy & Proffit (1985) [68]
**Bone-active drugs**	Produce the most significant effects on treatment duration and biomechanics	Bisphosphonates, PTH analogs, GLP-1 agonists
**Inflammation-modulating drugs**	Influence risk of root resorption and alter pain patterns	NSAIDs, corticosteroids, cytokine-modulating agents
**Topical antimicrobials and probiotics**	Improve oral/periodontal health but do not affect tooth movement	Chlorhexidine, red propolis, probiotics, MI Paste
**Systemic immunomodulators**	Introduce treatment complications and require individualized protocols	Cyclosporine, glucocorticoids, post-transplant drugs
**Orthodontic management considerations**	Force systems should be adapted; closer follow-ups recommended; adjunctive imaging and physician collaboration may be needed	Especially in long-term pharmacotherapy

## Data Availability

The data presented in this study are available upon reasonable request, after the signature of a formal data-sharing agreement in an anonymous form, from the corresponding authors.

## Supplementary Materials

The following supporting information can be downloaded at: https://www.mdpi.com/article/10.3390/bioengineering13020224/s1, Table S1: Effects of NSAIDs and analgesics on pain, inflammation, tooth movement, and root resorption during orthodontic treatment. The table includes animal studies; Table S2: Effects of NSAIDs and analgesics on pain, inflammation, tooth movement, and root resorption during orthodontic treatment. The table includes both clinical and preclinical studies, highlighting main findings and clinical relevance; Table S3: ROB of the studied involved; Table S4: Effects of drugs affecting bone metabolism. The table includes animal studies; Table S5: Effects of drugs affecting bone metabolism. The table includes both clinical and preclinical studies, highlighting main findings and clinical relevance; Table S6: ROB of the studied involved; Table S7: Effects of hormones and endocrine agents. The table includes animal studies; Table S8: Effects of hormones and endocrine agents. The table includes both clinical and preclinical studies, highlighting main findings and clinical relevance; Table S9: ROB of the studied involved; Table S10: Effects of chlorhexidine in orthodontic patients. The table includes both clinical and preclinical studies, highlighting main findings and clinical relevance; Table S11: Effects of probiotics, topical agents, and specialty toothpaste in orthodontic patients. The table includes both clinical and preclinical studies, highlighting main findings and clinical relevance; Table S12: ROB of the studied involved; Table S13: Effects of systemic special drugs and immunomodulators. The table includes animal studies; Table S14: Effects of systemic special drugs and immunomodulators. The table includes both clinical and preclinical studies, highlighting main findings and clinical relevance; Table S15: ROB of the studied involved; Table S16: Effects of other biological substances or indirect pharmacological approaches. The table includes both clinical and preclinical studies, highlighting main findings and clinical relevance; Table S17: ROB of the studied involved.

## Abbreviations

The following abbreviations are used in this manuscript:

OTM	Orthodontic Tooth Movement
NSAIDs	Non-Steroidal Anti-Inflammatory Drugs
PGE2	Prostaglandin E2
PGE1	Prostaglandin E1
PTH	Parathyroid Hormone
iPTH	Intermittent Parathyroid Hormone
RANKL	Receptor Activator of Nuclear Factor Kappa-B Ligand
OPG	Osteoprotegerin
PDL	Periodontal Ligament
GCF	Gingival Crevicular Fluid
STAT3	Signal Transducer and Activator of Transcription 3
AMPK	AMP-Activated Protein Kinase
NF-κB	Nuclear Factor Kappa-Light-Chain-Enhancer of Activated B Cells
GLP-1	Glucagon-Like Peptide 1
IL-1β	Interleukin 1 beta
IL-6	Interleukin 6
IL-17	Interleukin 17
TNF-α	Tumor Necrosis Factor alpha
IFN-γ	Interferon gamma
VAS	Visual Analog Scale
CPP-ACP	Casein Phosphopeptide–Amorphous Calcium Phosphate
CPP-ACFP	Casein Phosphopeptide–Amorphous Calcium Fluoride Phosphate
RCT	Randomized Controlled Trial
PRISMA	Preferred Reporting Items for Systematic Reviews and Meta-Analyses
PRISMA-P	Preferred Reporting Items for Systematic Review and Meta-Analysis Protocols
ROB	Risk of Bias
GRADE	Grading of Recommendations Assessment, Development and Evaluation
ADHD	Attention Deficit Hyperactivity Disorder
