# Supplementary material for: Influence of Pharmacological Agents on Orthodontic Tooth Movement: A Systematic Review
Source: Bioengineering (Basel). 2026 Feb 14;13(2):224. doi: 10.3390/bioengineering13020224 (PMC12938123; doi:10.3390/bioengineering13020224)
Supplement: Supplementary file 1 [file bioengineering-13-00224-s001.zip › bioengineering-4112940-supplementary.pdf]

Table S1. Effects of NSAIDs and analgesics on pain, inflammation, tooth movement, and root resorption during orthodontic treatment. The table includes animal studies.

Study [Author, Year]	Drug / Agent	Study Design / Model	Orthodontic Intervention / Dose	Outcomes Measured	Main Findings	Clinical Relevance
Kirschneck et al., 2017 [5]	Meloxicam	Animal study [rats] + in vitro	Systemic meloxicam	Tooth movement velocity, inflammatory root resorption	Reduced OTM velocity; significant decrease in root resorption; reduced osteoclast activity	Protective effect on dental tissues, but may slow treatment
Hu et al., 2015 [21]	Ibuprofen	Animal study [rats]	Systemic ibuprofen	RANKL, OPG, IGF-I expression; root resorption	Decreased RANKL, slight change in OPG; reduced osteoclastic activity; less root resorption	May slow tooth movement; protective against resorption
Tunçer et al., 2014 [22]	Various analgesics [NSAIDs, acetaminophen]	Animal study	Systemic analgesics	PGE2 levels in GCF	All analgesics reduced PGE2; ibuprofen most effective	Confirms anti-inflammatory effect; may impact tooth movement rate

Table S2. Effects of NSAIDs and analgesics on pain, inflammation, tooth movement, and root resorption during orthodontic treatment. The table includes both clinical and preclinical studies, highlighting main findings and clinical relevance.

Study [Author, Year]	Drug / Agent	Study Design / Model	Orthodontic Intervention / Dose	Outcomes Measured	Main Findings	Clinical Relevance
Shetty et al., 2013 [2]	Ibuprofen vs Acetaminophen	Clinical trial [humans]	Oral ibuprofen 400 mg vs acetaminophen 500 mg	PGE2 levels in GCF	Ibuprofen more effectively reduced PGE2 than acetaminophen	Supports ibuprofen for reducing inflammatory mediators during OTM
Salmassian et al., 2009 [17]		Clinical trial [humans]	Oral ibuprofen 400 mg vs acetaminophen 650 mg	Pain [VAS]	Ibuprofen superior to acetaminophen in early post-procedure pain control	Ibuprofen preferred analgesic for orthodontic pain
Gupta et al., 2014 [1]	NSAIDs	RCT, double-blind, placebo [humans]	Oral NSAID	Pain [VAS]	NSAIDs significantly reduced pain vs placebo	Effective pain management strategy
Minor et al., 2009 [18]	Preoperative Ibuprofen	Clinical trial [humans]	Single pre-op dose ibuprofen	Pain after separator placement	Reduced pain compared to placebo; effective preemptively	Preemptive analgesia recommended
Murdock et al., 2010 [19]	OTC analgesics vs bite-wafer	Noninferiority RCT [humans]	Ibuprofen / acetaminophen or bite-wafer	Pain	Analgesics noninferior to bite-wafer; early pain reduction	Supports use of analgesics as effective, convenient option
Hosseinzadeh Nik et al., 2016 [20]	Acetaminophen vs Liquefied Ibuprofen	Triple-blinded RCT [humans]	Oral acetaminophen vs liquefied ibuprofen	Pain during separation	Ibuprofen slightly more effective than acetaminophen	Supports ibuprofen preference in early pain
Kluemper et al., 2002 [23]	Benzocaine wax	Clinical trial [humans] – Topical analgesic	Topical benzocaine wax	Oral mucosal pain	Significant reduction in mucosal discomfort	Safe adjunct for appliance-

						induced soft tissue pain
Ngan et al., 1994 [24]	Ibuprofen single dose	Clinical trial [humans] – Topical / mucosal pain	Single-dose ibuprofen	Patient discomfort	Reduced discomfort at 2, 6, 24h post-archwire/separator	Confirms ibuprofen efficacy [localized analgesic, non-systemic OTM]

Abbreviations on table S1-S2: OTM, Orthodontic Tooth Movement; NSAIDs, Non-Steroidal Anti-Inflammatory Drugs; PGE2, Prostaglandin E2; GCF, Gingival Crevicular Fluid; RANKL, Receptor Activator of Nuclear Factor Kappa-B Ligand; OPG, Osteoprotegerin; IGF-I, Insulin-Like Growth Factor I; VAS, Visual Analog Scale; RCT, Randomized Controlled Trial; OTC, Over-The-Counter.

Table S3. ROB of the studied involved.

Meloxicam & orthodontic tooth movement [rats]	Kirschneck C, Meier M, Bauer K, Proff P, Fanghänel J., [5]	HIGH
Receptor activator of nuclear factor-kappa ligand, OPG, and IGF-I expression during orthodontically induced inflammatory root resorption in the recombinant human growth hormone-treated rats.	Hu Y, Liu W, Liu Z, Kuang W, He H. Angle Orthod. 2015. [10]	HIGH
Comparison of the effects of ibuprofen and acetaminophen on PGE_2 levels in the GCF during orthodontic tooth movement.	Shetty N, Patil AK, Ganeshkar SV, Hegde S. Prog Orthod. 2013. [2]	HIGH
Effects of various analgesics on the level	Tunçer Z, Polat-Ozsoy O, Demirbilek M,	HIGH

of prostaglandin E_2 during orthodontic tooth movement.	Bostanoğlu E. Eur J Orthod. 2014. [22]	
Comparison of the efficacy of ibuprofen and acetaminophen in controlling pain after orthodontic tooth movement.	Salmassian R, Oesterle LJ, Shellhart WC, Newman SM. Am J Orthod Dentofacial Orthop. 2009. [17]	MODERATE
Controlling pain during orthodontic fixed appliance therapy with NSAIDs: randomized, double-blinded, placebo-controlled study.	Gupta M, Kandula S, Laxmikanth SM, Vyavahare SS, Reddy SB, Ramachandra CS. J Orofac Orthop. 2014. [1]	MODERATE
Effects of preoperative ibuprofen on pain after separator placement.	Minor V, Marris CK, McGorray SP, Yezierski R, Fillingim R, Logan H, Wheeler TT. Am J Orthod Dentofacial Orthop. 2009. [18]	HIGH
Treatment of pain after initial archwire placement: noninferiority RCT [OTC analgesics vs bite-wafer].	Murdock S, Phillips C, Khondker Z, Hershey HG. Am J Orthod Dentofacial Orthop. 2010. [19]	HIGH
The effect of ibuprofen [and aspirin] on orthodontic discomfort — single-dose randomized trial.	Ngan P. et al. Am J Orthod Dentofacial Orthop. 1994. [24]	MODERATE
Acetaminophen versus liquefied ibuprofen for control of pain during separation in	Hosseinzadeh Nik T., 2016 [20]	MODERATE

orthodontic patients [triple-blinded RCT].		
Efficacy of a wax containing benzocaine in relief of oral mucosal pain caused by orthodontic appliances.	Kluemper GT., 2022 [23]	MODERATE

Table S4. Effects of drugs affecting bone metabolism. The table includes animal studies.

**Study [Author, Year]**	**Drug /Agent**	**Study Design / Model**	**Intervention/ Dose**	**Main Outcome**	**Biological Mechanism**	**Effect on OTM**	**Clinical Relevance**
Zhang et al., 2020 [9]	**Parathyroid Hormone [PTH]**	Rat model with periodontitis	Intermittent systemic PTH	Increased alveolar bone homeostasis, reduced bone loss	STAT3 and β-catenin activation, osteoblast stimulation	Accelerates/ supports	Supports OTM in compromised bone conditions, improves bone stability
Li et al., 2019 [25]		Rat post-osteotomy	Recombinant human PTH	Accelerated tooth movement	Enhanced bone remodeling	Accelerates	Reduces treatment time after surgical interventions
Lee et al., 2004 [28]	**Bisphosphonates**	Rat model	Bisphosphonate treatment	Reduced osteoclast activity, slower bone resorption	Anti-resorptive, inhibits osteoclasts	Inhibits	May require longer treatment and careful force application

Shen et al., 2021 [29]	**Exendin-4**	Mouse model	Local high-dose exendin-4	Reduced osteoclast number, decreased RANKL and TNF-α	Anti-osteoclastic, immunomodulatory	Inhibits	Potential impact in diabetic patients on GLP-1 agonists
Xu et al., 2021 [10]	**Simvastatin**	Rat model	Local simvastatin	Reduced inflammation, preserved anchorage	AMPK/MAPK/NF-κB inhibition, reduced osteoclastogenesis	Neutral / modulatory	Useful to maintain anchorage; does not accelerate OTM
Rajasekaran & Nayak, 2014 [32]	**Prostaglandin E1 [PGE1]**	Rat model	PGE1 injection	Increased OTM and osteoclast activity	Enhanced bone remodeling	Accelerates	Preclinical support for accelerated OTM strategies

Table S5. Effects of drugs affecting bone metabolism. The table includes both clinical and preclinical studies, highlighting main findings and clinical relevance.

**Drug /Agent**	**Study [Author, Year]**	**Study Design / Model**	**Intervention/ Dose**	**Main Outcome**	**Biological Mechanism**	**Effect on OTM**	**Clinical Relevance**
**Parathyroid Hormone [PTH]**	Charavet et al., 2016 [26]	Adult humans, RCT	Local PTH + piezoelectric alveolar decortication	Enhanced tooth movement	Local bone remodeling	Accelerates	Potential adjunct in adult orthodontics post-surgery
**Bisphosphonates**	Lotwala et al., 2012 [27]	Human, retrospective cohort	Chronic bisphosphonate therapy	Reduced tooth movement, harder to achieve planned displacement	Osteoclast inhibition	Inhibits	High risk of suboptimal outcomes; monitor bone remodeling

**Osteoprotegerin [OPG]**	Li & Tang, 2009 [30]	Conceptual	Local OPG delivery	Theoretical blockade of osteoclast-mediated resorption	RANKL antagonism	Inhibits	May reinforce anchorage but slow tooth movement; conceptual only
**Prostaglandin E1 [PGE1]**	Yamasaki et al., 1984 [31]	Human clinical	Local PGE1 application	Increased tooth movement	Osteoclast activation	Accelerates	Can reduce treatment time but may increase root resorption risk
**Clinical algorithm / drug burden**	Tsvetkova & Kovalenko, 2023 [33]	Human / narrative	Review of patients with drugs reducing bone density	Adjusted orthodontic protocols	N/A	N/A	Guides treatment planning in patients on osteometabolic drugs

Table S6. ROB of the studied involved.

			Main reasons for ROB judgement
Local delivery of simvastatin maintains tooth anchorage	Xu L et al., 2021 [10]	HIGH	**High risk of bias**, mainly due to unclear randomization procedures, lack of allocation concealment, absence of blinding of operators and outcome assessors, and potential selective reporting in an animal experimental model.
Local administration of exendin-4 inhibits	Shen WR et al., 2021 [29]	HIGH	**High risk of bias**, mainly due to unclear randomization procedures, lack of

orthodontic tooth movement			allocation concealment, absence of blinding of operators and outcome assessors, small sample size, and selective outcome reporting in an animal experimental model.
Accelerated tooth movement with rhPTH	Li Y et al., 2019 [25]	HIGH	Lack of blinding and allocation concealment<br><br>Insufficient reporting of attrition and handling of missing data<br><br>Potential performance bias due to experimental design
Local delivery of osteoprotegerin	Li Y & Tang L, 2009 [30]	not applicable	This article is a hypothesis-driven conceptual paper without experimental design, comparator groups, or outcome assessment; therefore, risk of bias evaluation is not applicable.
Continuous vs interrupted orthodontic force	Lee KJ et al., 2004 [28]	MODERATE	**Moderate risk of bias**, mainly due to unclear randomization of the split-mouth allocation, lack of blinding of operators and outcome assessors, despite a

			well-controlled experimental design and objective biochemical outcome measures.
Bisphosphonates & orthodontic outcomes	Lotwala RB et al., 2012 [27]	HIGH	**High risk of bias**, mainly due to the retrospective observational design, lack of randomization, selection bias, uncontrolled confounding variables, and absence of blinding in outcome assessment.<br><br>Confounding factors not adequately controlled
Localized Piezoelectric alveolar decortication	Charavet C et al., 2016 [26]	MODERATE	**Moderate risk of bias**, mainly due to lack of blinding of participants and operators and unclear blinding of outcome assessors, despite adequate randomization, baseline comparability, and trial registration.
Clinical application of prostaglandin E1 [PGE1]	Yamasaki K et al., 1984 [31]	HIGH	**High risk of bias**, mainly due to lack of randomization and allocation concealment, absence of blinding, non-standardized outcome assessment, and incomplete

			methodological reporting.
Prostaglandin E1 vs corticotomy	Rajasekaran UB & Krishna Nayak US, 2014 [32]	MODERATE	**Moderate risk of bias**, mainly due to unclear randomization of the split-mouth allocation, lack of blinding of participants, operators, and outcome assessors, despite an appropriate intra-subject comparative design.
Orthodontic treatment algorithm – drugs reducing bone mineral density	Tsvetkova M & Kovalenko A, 2023 [33]	not applicable	**Not applicable** – This publication presents a clinical treatment algorithm and descriptive application in patients with drug-related risk factors, without a comparative study design or outcome assessment suitable for risk of bias evaluation.
Parathyroid hormone increases alveolar bone homoeostasis during orthodontic tooth movement in rats with periodontitis	Zhang C. et al., 2020 [9]	HIGH	**High risk of bias**, mainly due to unclear randomization and allocation concealment, lack of blinding of operators and outcome assessors, and potential selective reporting in an animal experimental model.

Table S7. Effects of hormones and endocrine agents. The table includes animal studies.

Drug / Agent	Study [Author, Year]	Study Design / Model	Orthodontic Intervention / Dose	Outcomes Measured	Main Findings	Clinical Relevance
Vitamin E	Seong et al., 2022 [34]	Animal study [rats]	Oral vitamin E diet	OTM rate, osteoclast/osteoblast activity	Increased OTM; enhanced bone remodeling	Vitamin E may act as an adjuvant to accelerate movement
Vitamin D	Iosub Ciur et al., 2016 [37]	Animal study [rats]	Local vitamin D injection	OTM rate, bone remodeling markers	Accelerated OTM; increased osteoblast activity	Vitamin D supplementation may optimize bone remodeling

Table S8. Effects of hormones and endocrine agents. The table includes both clinical and preclinical studies, highlighting main findings and clinical relevance.

Drug / Agent	Study [Author, Year]	Study Design / Model	Orthodontic Intervention / Dose	Outcomes Measured	Main Findings	Clinical Relevance
Sex hormones	Peruga & Lis, 2024 [11]	Prospectiv e cohort [humans]	Observation of natural hormone levels	OTM rate, alveolar bone changes	Testosterone associated with faster OTM; estrogen modulated movement	Hormonal status should inform treatment duration and force selection
Sex hormones	Quast et al., 2021 [40]	In vitro [human PDL cells]	Mechanical stretch ± hormone receptors	Osteogenic markers, receptor expression	Donor variation in receptor expression affects osteogenesis	Individual endocrine profiles may influence bone remodeling

Vitamin E	Esenlik et al., 2012 [35]	Clinical trial [humans]	Oral supplementation	GCF oxidative stress markers	Reduced lipid peroxidation; improved antioxidant status	Supports anti-inflammatory and bone-protective effect
Vitamin D	Tashkandi et al., 2021 [36]	Longitudinal cohort [humans]	Salivary biomarker monitoring	Vitamin D binding protein, bone turnover markers	Correlation between vitamin D and bone metabolism	Vitamin D status may guide orthodontic planning
Glucocorticoids	Tsvetkova, 2022 [38]	Clinical analysis + systematic review	Chronic glucocorticoid use	Bone mineral density, treatment complications	Reduced bone density; higher caries/xerostomia risk	Tailored orthodontic algorithm recommended
Nicotine	Yu et al., 2018 [39]	In vitro [human PDL cells]	Cyclic tensile stress ± nicotine	Osteogenic differentiation, Wnt pathway	Nicotine inhibited osteogenesis; downregulated Wnt signaling	Smoking may slow OTM and impair bone remodeling
Steroid hormones	Buchhardt et al., 2022 [41]	Clinical observation [humans]	Hormone measurement	Tooth wear, bone metabolism	Steroid hormones correlated with increased tooth wear	Hormone levels may influence enamel/bone remodeling

Table S9. ROB of the studied involved.

Correlation of sex hormone levels with orthodontic tooth movement in the maxilla	Peruga 2024 [11]	HIGH

Vitamin E enriched diet increases the rate of orthodontic tooth movement	Seong 2022 [34]	HIGH
Vitamin E supplementation modulates GCF lipid peroxidation	Esenlik 2012 [35]	HIGH
Influence of local Vitamin D on rate of OTM	Iosub Ciur 2016 [37]	HIGH
Longitudinal assessment of salivary Vit D binding protein	Tashkandi 2021 [36]	HIGH
Orthodontic treatment algorithm for patients with positive drug anamnesis. Glucocorticoids	Tsvetkova 2022 [38]	NOT APPLICABLE
Nicotine inhibits osteogenic differentiation of hPDLCs	Yu 2018 [39]	NOT APPLICABLE
Influence of Steroid Hormones on Tooth Wear	Buchhardt 2022 [41]	HIGH
Donor variation & sex hormone receptors in PDL cells	Quast 2021 [40]	NOT APPLICABLE

Table S10. Effects of chlorhexidine in orthodontic patients. The table includes both clinical and preclinical studies, highlighting main findings and clinical relevance.

Drug / Formulation	Study [Author]	Study Design	Orthodontic Intervention / Dose	Outcomes Measured	Main Findings	Clinical Relevance
**Chlorhexidine 0.2% mouthrinse**	**Akhtar et al. [2022]** [42]	Randomized controlled trial	0.2% CHX mouthrinse adjunct for 2 weeks	PI, BOP, PPD, CAL, MN genotoxicity	PI & BOP ↓; PPD & CAL =; MN ↑	Effective plaque control; possible genotoxicity risk
**Chlorhexidine varnish 40%**	**Jenatschke et al. [2001]** [43]	Longitudinal RCT	40% CHX varnish every 8 weeks during treatment	Salivary MS; DMFS increment	Temporary MS suppression; caries increment similar to placebo	Limited long-term benefit; temporary antibacterial effect
**Chlorhexidine varnish [Cervitec]**	**Madléna et al. [2000]** [44]	Split-mouth, longitudinal	Cervitec varnish every 3 months	Plaque SM, salivary SM/LB; new lesions	SM in plaque ↓; caries in treated quadrant ↓	Effective localized caries-prevention around brackets
**Chlorhexidine varnish [EC40]**	**Pretti et al. [2015]** [45]	Randomized split-mouth	EC40 CHX varnish single application	Gingival volume via digital measurement	Significant gingival overgrowth reduction	Useful for reducing gingival enlargement in fixed appliances
**Chlorhexidine-containing varnish**	**Paschos et al. [2008]** [46]	Randomized clinical split-mouth	CHX varnish at 0, 12, 24 weeks	PD, GI, PI, GCF IL-1β	GI & PI ↓ in varnish sites	Helps control inflammation around orthodontic attachments

**Chlorhexidine mouthrinse 0.12%**	**Anderson et al. [1997]** [47]	Randomized controlled trial	0.12% CHX for 3 months	PI, GI, RI, DI, PD	PI & GI ↓ significantly; DI ↑ slightly	Effective for plaque/gingivitis reduction; staining possible
**Chlorhexidine treatments**	**Lundström & Krasse [1987]** [48]	Prospective clinical study	CHX treatments before & during treatment	Salivary SM & LB	SM ↓ significantly; rapid recolonization; LB unaffected	Useful short-term antibacterial effect; requires monitoring
**Persica herbal mouthwash vs CHX 0.2%**	**Salehi & Momeni Danaie [2006]** [49]	Randomized clinical trial	Persica vs 0.2% CHX mouthwash	SM counts; side effects	CHX more potent; Persica fewer side effects	Herbal alternative with lower efficacy but better tolerance

Table S11. Effects of probiotics, topical agents, and specialty toothpaste in orthodontic patients. The table includes both clinical and preclinical studies, highlighting main findings and clinical relevance.

Drug / Agent	Study [Author, Year]	Study Design / Model	Orthodontic Intervention / Dose	Outcomes Measured	Main Findings	Clinical Relevance
Amine fluoride / stannous fluoride	Øgaard et al., 2006 [54]	RCT, humans	AmF/SnF_2 toothpaste + mouthrinse	Plaque, gingivitis, white spot lesions	Lower increase of WSL, plaque and gingivitis vs NaF	Effective preventive strategy during fixed orthodontics
Fluoride [water exposure]	Karadeniz et al., 2011 [55]	Clinical study, humans	Environmental fluoride [0.05 vs 2 ppm]	Tooth movement [2D/3D]	Fluoride concentration influenced orthodontic tooth movement	Relevant for biomechanics in high-

						fluoride areas
Fluoride varnish	Farhadian et al., 2008 [56]	In vivo split-mouth	Fluoride varnish around brackets	Enamel demineralization depth	Significantly reduced demineralization	Effective protection of enamel around brackets
Fluoride varnish	Gontijo et al., 2007 [57]	In vivo study	Single Duraphat® application	Enamel Ca, P, F content	Higher mineral content around brackets	Supports topical fluoride use during treatment
Antimicrobial + fluoride varnish	Øgaard et al., 2001 [58]	RCT, humans	Combined varnish application	WSL, plaque, gingivitis	Trend toward fewer new WSL on incisors	Adjunctive preventive option
Fluoride varnish	Vivaldi-Rodrigues et al., 2006 [59]	Prospective clinical study	Repeated varnish every 3 months	White spot lesions	44% less demineralization vs control	Clinically useful for WSL prevention
High-dose topical fluoride	Alexander & Ripa, 2000 [60]	Clinical trial, humans	5000-ppm fluoride gel/dentifrice	Enamel demineralization	Significant reduction vs standard fluoride	Supports intensive fluoride regimens
Low-fluoride regimen	Willmot, 2004 [61]	RCT, humans	Low-fluoride toothpaste/mouth rinse	White lesion size and regression	Lesion reduction over time	Fluoride level influences post-treatment recovery

CPP-ACFP paste	Beerens et al., 2010 [62]	RCT, humans	CPP-ACFP paste, 3 months	WSL remineralization [QLF]	No advantage vs control	No added benefit beyond normal hygiene
MI Paste Plus	Beerens et al., 2018 [13]	RCT, 12-month follow-up	Daily topical application	Lesion fluorescence, area	No difference vs placebo	Not superior for post-orthodontic lesions
Chlorhexidine/thymol varnish	Sköld-Larsson et al., 2004 [63]	Split-mouth clinical study	Varnish every 6 weeks	Fissure caries [LF]	Reduced caries progression	Protective adjunct during orthodontics
Brazilian Red Propolis dentifrice	Furtado Júnior et al., 2020 [64]	RCT, humans	Dentifrice twice daily	GBI, microbial load	Reduced gingival bleeding and CFU	Safe adjunct for gingival control
Probiotic drops [L. reuteri]	Alforaidi et al., 2021 [52]	RCT, humans	Daily probiotic drops	Plaque pH, S. mutans	Reduced acidogenicity	Adjunctive microbial control
Probiotics [systemic vs topical]	Alp & Baka, 2018 [53]	Clinical trial, humans	Kefir or probiotic toothpaste	Salivary bacteria	Reduced S. mutans and Lactobacillus	Supports probiotic use
H_2O_2 1.5% + NaF rinse	Boyd, 1989 [65]	Clinical trial, humans	Daily rinse	Plaque, gingivitis	Significant gingival improvement	Simple adjunct for periodontal health

Table S12. ROB of the studied involved.

Comparative assessment of periodontal status and genotoxicity [chlorhexidine mouthrinse]	Akhtar et al., 2022 [42]	High
Effect of chlorhexidine varnish on gingival growth	Pretti et al., 2015 [45]	Moderate/Low
Orthodontic attachments and CHX varnish effects on gingival health	Paschos et al., 2008 [46]	High
Clinical effects of chlorhexidine mouthwashes	Anderson et al., 1997 [47]	High
Influence of repeated CHX varnish on mutans streptococci & caries	Jenatschke et al., 2001 [43]	High
Effect of CHX varnish on bacterial levels in plaque and saliva	Madléna et al., 2000 [44]	Moderate
Streptococcus mutans and lactobacilli frequency in orthodontic patients; effect of chlorhexidine treatments	Lundström & Krasse, 1987 [48]	High
Antibacterial effects of Persica vs chlorhexidine on Streptococcus mutans	*Salehi P. et al, 2006 [49]*	*High*
Effects of AmF/SnF_2 toothpaste/mouthrinse	Øgaard et al., 2006 [54]	Moderate

on plaque, gingivitis, WSL		
Effect of fluoride on orthodontic tooth movement	Karadeniz et al., 2011 [55]	High
Effect of fluoride varnish on enamel demineralization around brackets	Farhadian et al., 2008 [56]	Moderate
Dental enamel around fixed orthodontic appliances after fluoride varnish application	Gontijo et al., 2007 [57]	High
Effects of combined application of antimicrobial and fluoride varnishes in orthodontic patients	Øgaard et al., 2001 [58]	Moderate
The effectiveness of a fluoride varnish in preventing the development of white spot lesions	Vivaldi-Rodrigues et al., 2006 [59]	Moderate
Effects of Self-Applied Topical Fluoride Preparations in Orthodontic Patients	Alexander & Ripa, 2000 [60]	High
White lesions after orthodontic treatment: Does low fluoride make a difference?	Willmot, 2004 [61]	Moderate
Effects of CPP-ACFP paste on white spot lesions	Beerens et al., 2010 [62]	Moderate/Low

Long-term remineralizing effect of MI Paste Plus	Beerens et al., 2018 [13]	Low
Effect of chlorhexidine/thymol varnish on fissure caries	Sköld-Larsson et al., 2004 [63]	High
Brazilian Red Propolis dentifrice in orthodontic patients	Furtado Júnior et al., 2020 [64]	Moderate
Effect of Lactobacillus reuteri drops on plaque acidogenicity	Alforaidi et al., 2021[52]	Moderate
Effects of probiotics on salivary S. mutans and Lactobacillus	Alp & Baka, 2018 [53]	High
Effects on gingivitis of daily rinsing with 1.5% H_2O_2	Boyd, 1989 [65]	High

Table S13. Effects of systemic special drugs and immunomodulators. The table includes animal studies.

**Drug / Agent**	**Study [Author, Year]**	**Study Design / Model**	**Intervention / Dose**	**Outcomes Measured**	**Main Findings**	**Clinical Relevance**
Methylphenidate	Aghili et al., 2017 [69]	Animal study [rats]	Systemic methylphenidate	Tooth movement, bone histology	Reduced OTM; altered bone microarchitecture	Suggests caution in patients on chronic methylphenidate therapy

Table S14. Effects of systemic special drugs and immunomodulators. The table includes both clinical and preclinical studies, highlighting main findings and clinical relevance.

Drug / Agent	Study [Author, Year]	Study Design / Model	Intervention / Dose	Outcomes Measured	Main Findings	Clinical Relevance
Cyclosporine	Daley et al., 1991 [12]	Case report / human	Patient on systemic cyclosporine	Tooth movement, gingival response	Gingival hyperplasia observed; potential altered tooth movement	May complicate orthodontic treatment; monitor gingival tissues closely
Thymosin α1	Loo et al., 2008 [66]	Double-blind RCT / human	Local Tα1 application on avulsed teeth	Periodontal healing, cytokine levels, ankylosis, tooth movement	Enhanced healing; lower IFN, TNF-α, IL-6; reduced ankylosis; better tooth stability	Supports Tα1 for improved outcomes in tooth reimplantation
Phenobarbital	Livingston et al., 1980 [71]	Observational / human	Oral phenobarbital vs phenytoin	Bone health, seizure control	Potential indirect effects on bone; no direct OTM data	Consider bone metabolism effects in epilepsy patients
Immunosuppressants post-transplant	Senirkentli et al., 2025 [67]	Clinical / children	Systemic immunosuppressive therapy	Oral health status, periodontal health	Increased gingival overgrowth, periodontal inflammation	Monitor oral health closely; adapt orthodontic interventions

Medically compromised patients	van Venrooy & Proffit, 1985 [68]	Observational / human	Various medical conditions	Tooth movement, treatment feasibility	Treatment possible with modified biomechanics	Individualized orthodontic planning required
Corticosteroids in juvenile arthritis	Kjellberg, 1998 [70]	Cohort study / human	Long-term corticosteroid therapy	Craniofacial growth, tooth movement	Reduced mandibular/maxillary growth; altered craniofacial patterns	Plan treatment considering growth alterations

Table S15. ROB of the studied involved.

Orthodontic therapy in the patient treated with cyclosporine	Daley, Wysocki & Mamandras, 1991 [12]	HIGH
Thymosin alpha 1 provides short-term and long-term benefits in the reimplantation of avulsed teeth	Loo et al., 2008 [66]	MODERATE
Phenobarbital vs. phenytoin for grand mal epilepsy	Livingston et al., 1980 [71]	HIGH
Effect of methylphenidate on orthodontic tooth movement and histological features of bone tissue in rats	Aghili et al., 2017 [69]	HIGH
Oral Health Status of Children With a History of Liver Transplantation	Senirkentli, Polat & Onder, 2025 [67]	HIGH

Orthodontic care for medically compromised patients: possibilities and limitations	van Venrooy & Proffit, 1985 [68]	not applicable
Craniofacial growth in juvenile chronic arthritis	Kjellberg, 1998 [70]	HIGH

Table S16. Effects of other biological substances or indirect pharmacological approaches. The table includes both clinical and preclinical studies, highlighting main findings and clinical relevance.

Drug / Agent / Mediator	Study [Author, Year]	Study Design / Model	Orthodontic Intervention / Dose	Outcomes Measured	Main Findings	Clinical Relevance
General medications	Turpin, 2009 [72]	Systematic literature review	N/A	Tooth movement rate, adverse effects	Hormones [estrogens], corticosteroids, bisphosphonates can decrease OTM; NSAIDs and some anti-inflammatory drugs can modulate OTM	Clinicians should review patient medications and anticipate altered tooth movement; adjust force and treatment duration
IL-1β & PGE	Saito, 1991 [73]	In vivo [cats] + in vitro PDL fibroblasts	Orthodontic force 80 gm on canine for 12h–7d; in vitro mechanical stress ± IL-1β	PGE, IL-1β expression; bone resorption [45Ca release]	Mechanical stress increased PGE & IL-1β; IL-1β enhanced bone resorption; indomethacin partially inhibited resorption	Cytokines mediate OTM-induced bone remodeling; potential for anti-inflammatory interventions

IL-17	Nakano et al., 2015 [74]	In vitro [dental pulp cells]	Orthodontic force simulation	IL-17 expression ; osteoclast activity; root resorption	IL-17 upregulated by force; increases osteoclast differentiation; blocking IL-17 reduces resorption	Targeting IL-17 may protect against root resorption during orthodontic treatment

Table S17. ROB of the studied involved.

Medications weigh-in on tooth movement	Turpin, 2009 [72]	not applicable
Interleukin-1β and prostaglandin E response to mechanical stress	Saito, 1991 [73]	High
Interleukin-17 in orthodontically induced inflammatory root resorption	Nakano et al., 2015 [74]	High
